# A Novel Combination of Metachronous Primary Malignancies of the Thyroid and Breast in a Patient with Neurofibromatosis Type 1

**DOI:** 10.7759/cureus.7590

**Published:** 2020-04-08

**Authors:** Dawood Findakly, Anup Solsi, Waqas Arslan

**Affiliations:** 1 Internal Medicine, Creighton University Arizona Health Education Alliance/Valleywise Health Medical Center, Phoenix, USA; 2 Hematology and Oncology, Creighton University Maricopa Medical Center, Phoenix, USA

**Keywords:** neurofibromatosis 1, papillary thyroid cancer, invasive ductal breast cancer, oncology

## Abstract

Neurofibromatosis 1 (NF1) is a genetic condition of variable presentations. It has been shown to increase the risk of multiple cancers. Therefore, NF1 has been identified as a tumor-provoking condition.

We present a case of a 39-year-old woman with NF1 who was diagnosed initially with papillary thyroid carcinoma (PTC) and subsequently presented with a painful breast lump. Core biopsy revealed an invasive ductal carcinoma (IDC) for which selective estrogen receptor modulator (SERM) therapy was initiated. A lumpectomy was performed soon after, which confirmed IDC. Following surgery, the patient received a combination of anthracycline and cyclophosphamide (AC), which was later followed by a taxol-based chemotherapy regimen.

This study aims to throw light on the rare phenomenon of metachronous malignancy: the occurrence of successive primary cancers in the same patient. We believe that raising awareness regarding the different neoplasms associated with NF1 is important to promote appropriate preemptive screening for early detection of a second primary neoplasm, which can help lower the morbidity and mortality associated with this condition through expedited intervention.

## Introduction

Neurofibromatosis 1 (NF1), also known as von Recklinghausen’s disease, is an autosomal dominant condition caused by a mutation in the neurofibromin-1 gene found on chromosome 17q11.2. NF1 has been linked to both benign and malignant tumors, including those of the eye, such as Lisch nodules and optic gliomas, as well as brain tumors, rhabdomyosarcoma, and some leukemias [1,2]. It is not surprising, therefore, that NF1 has also been shown to increase the risk of invasive carcinoma of the breast [3]. Unfavorable prognostic factors including negative estrogen receptor (ER), progesterone receptor (PR), and positive human epidermal growth factor receptor 2 (HER2) were previously reported in patients with NF1 who developed breast cancer [4].

Increased instances of non-medullary thyroid carcinoma in patients with NF1 have been described in the literature [5]. However, NF1 association with papillary thyroid carcinoma (PTC), one of the most common endocrine malignancies, is exceedingly rare [6].

Here we present an extremely unusual occurrence of two metachronous neoplasms in a 39-year-old multiparous woman, in whom NF1 was diagnosed first with PTC followed by invasive ductal carcinoma (IDC) of the breast years later. To the best of our knowledge, this is the first case to report this specific combination of metachronous primary tumors.

## Case presentation

**Figure 1:**
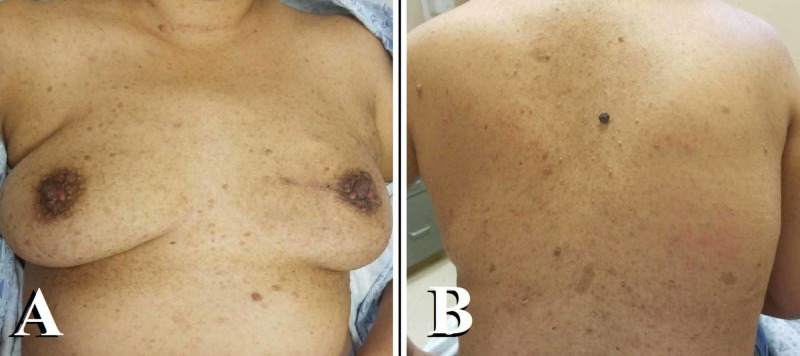
Multiple cutaneous neurofibromas A: front - the image shows multiple neurofibromas in the nipple-areolar region of both the breasts; B: back - the image shows the presence of multiple café au lait spots

**Figure 2:**
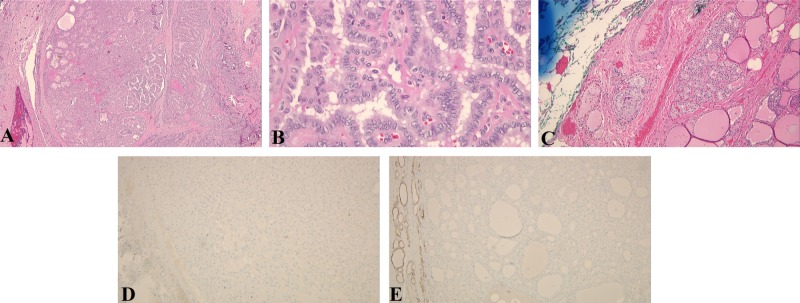
Sections from thyroid tissue showing PTC exhibiting papillary structures lined by cuboidal epithelial cells with enlarged nuclei with nuclear crowding, fine or clear chromatin, and small nucleoli A: H&E, x40; B: H&E, x400; C: H&E, x100 showing multiple small papillary microcarcinomas; D: immunoperoxidase staining negative for HBME-1; E: immunoperoxidase staining negative for CK19 PTC: papillary thyroid carcinoma; CK19: cytokeratin 19; H&E: hematoxylin and eosin

A 39-year-old G4P3 Hispanic woman with a past medical history of NF1 diagnosed in July 2003 was initially seen in September 2009 for thyroid evaluation (Figure 1). At that time, she underwent a thyroid biopsy, which was benign. She presented again in 2012 with dysphagia and was found to have a multinodular disease on thyroid ultrasound. A subsequent biopsy reported a benign tumor. Unfortunately, she was lost to follow up from 2015, until she was seen again in January 2018, when she underwent fine-needle aspiration (FNA) with biopsy-proven PTC (Figure 2).

**Figure 3:**
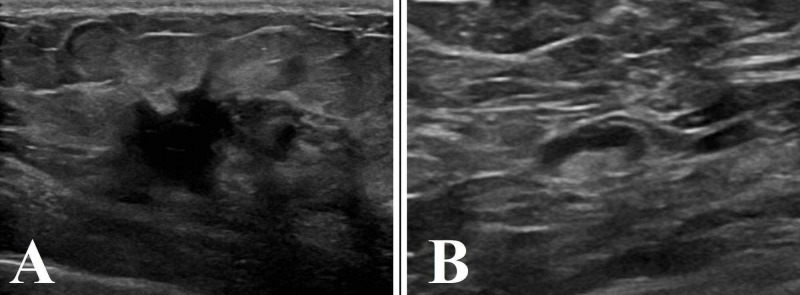
Breast ultrasound A: left breast showing a 2.6 x 1.4 x 1.9 cm irregular mass with speculated margins; B: ipsilateral axilla showing one abnormal appearing LN with thickened cortex measuring 4 mm LN: lymph node

In March 2019, she presented to her primary care physician complaining of six weeks of a painful left breast mass. The patient stated that her menarche had been at the age of 13 years, and her first pregnancy had occurred at the age of 23 years. She denied any family history of breast cancer. On examination, there were multiple cutaneous neurofibromas over the bilateral breast and trunk. A smooth, 0.5-cm lump was palpated at the 9 o’clock margin of the right areola. The ultrasound showed an irregular mass with speculated margins and a surrounding hyperechoic halo measuring 2.6 x 1.4 x 1.9 cm (Figure 3A). This mass was located 6 cm from the nipple. An abnormal appearing lymph node with a thickened cortex measuring 4 mm was also identified (Figure 3B).

**Figure 4:**
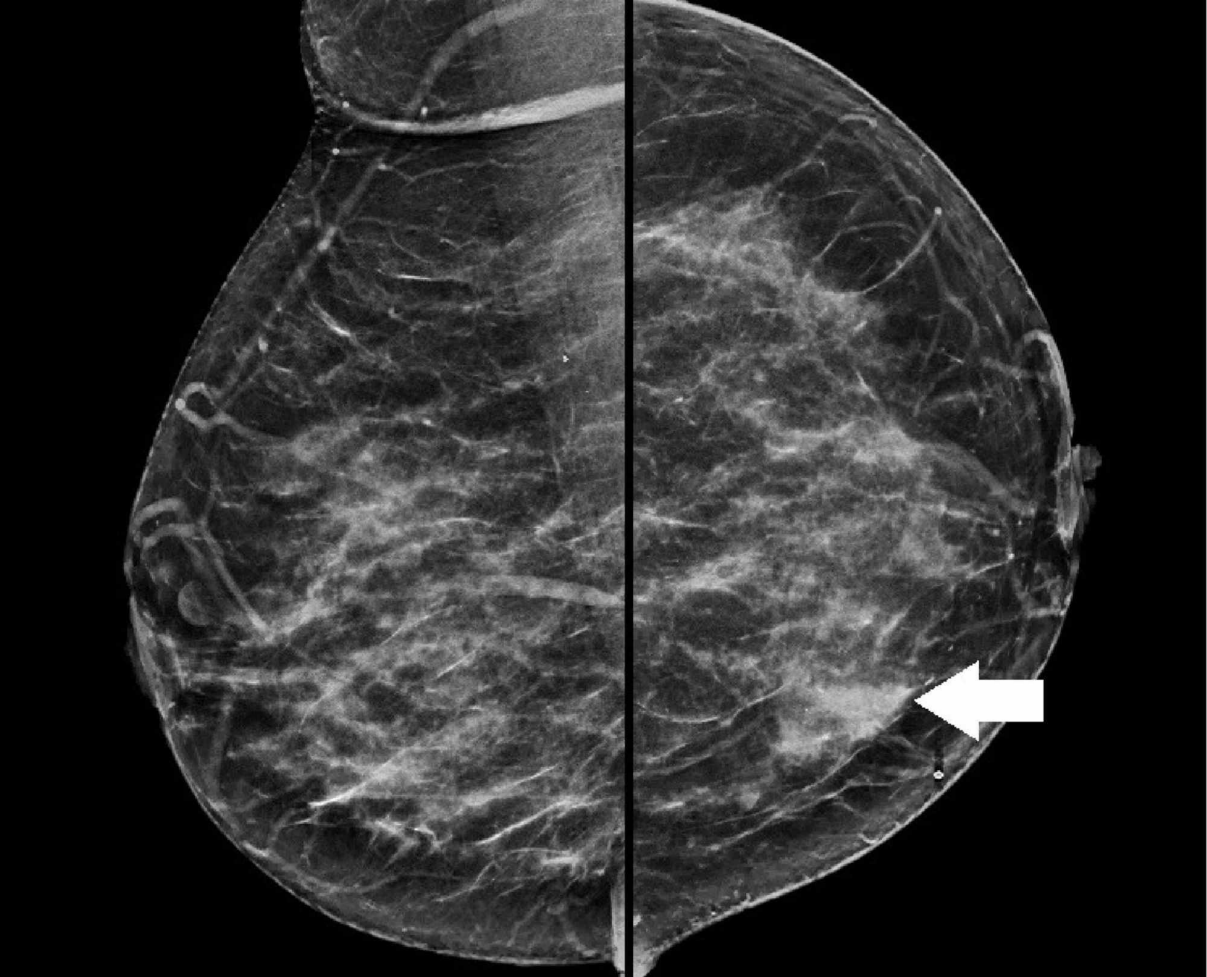
Diagnostic mammogram The image shows a solitary speculated mass (white arrow) measuring approximately 2.5 x 2.0 cm within the central inferior left breast with a subtle area of architectural distortion

Mammography was performed and it demonstrated a solitary, speculated mass measuring approximately 2.5 x 2.0 cm within the central inferior left breast with a subtle area of architectural distortion (Figure 4). Subsequently, an ultrasound-guided core biopsy was done, and histological diagnosis of a poorly differentiated, grade III left breast IDC was made.

**Figure 5:**
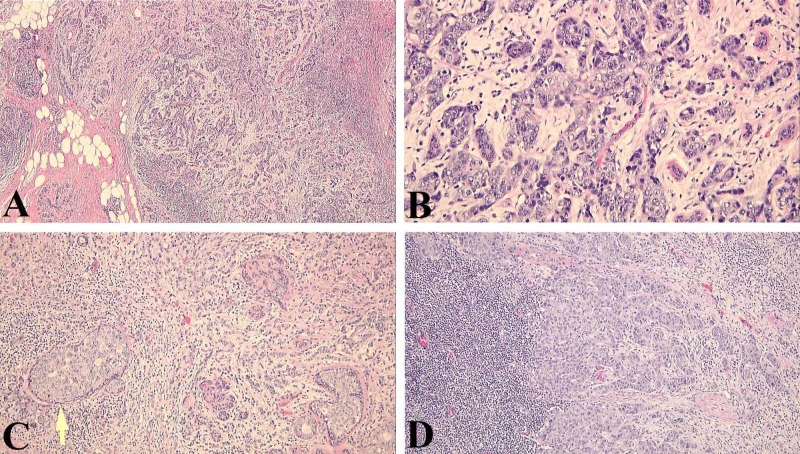
Tumor sections revealing the histopathological features of an IDC. Nests and clusters of cells with scant cytoplasm and enlarged hyperchromatic nuclei with moderate nuclear pleomorphism, nucleoli, and increased mitotic activity A: H&E, x40; B: H&E, x200; C: focal lymphatic invasion identified (yellow arrow) - H&E, x100; D: lymph node tissue with metastatic carcinoma - H&E, x100 IDC: invasive ductal carcinoma; H&E: hematoxylin and eosin

Serum markers from breast tissue biopsy revealed 90% ER positivity, 80% PR positivity, negative HER2-neu, and Ki-67 of 50% by immunohistochemistry (IHC). The patient was then referred to hematology and oncology for initiation of neo-adjuvant selective estrogen receptor modulator (SERM) therapy with tamoxifen. Left lumpectomy with sentinel lymph node (SLN) biopsy was performed eight weeks after the initial diagnosis, and a 2.3-cm mass was excised. Histopathology established the diagnosis of IDC with evidence of metastasis in 2/4 lymph nodes (Figure 5).

The gene expression profiling results reported an Oncotype Recurrence Score (RS) of 45, with an expected estimate of recurrence at nine years following adjuvant five-year hormonal therapy to be 37%. Following lumpectomy, the patient received adjuvant chemotherapy with adriamycin and cyclophosphamide for four cycles and is currently on paclitaxel therapy.

## Discussion

Patients with NF1 can present with a wide array of clinical manifestations, such as café au lait spots, axillary freckling, Lisch nodules, and neurofibromas, among others [3]. NF1 is known as a cancer-predisposing condition. Individuals with NF1 are estimated to have a two-fold increased lifetime risk of benign and/or malignant neoplasm compared to the general population. Due to this elevated cancer risk, it is reported that NF1 results in worsened survival outcomes [7,8]. 

The molecular mechanism behind the increased tumorigenesis in NF1 is related to mutations in the neurofibromin gene. Neurofibromin is a protein encoded by the NF1 gene that regulates the proliferation and differentiation of cells. Moreover, it is a tumor suppressor gene that is believed to play a significant role in regulating cellular levels of RAS proteins. Therefore, when modified, such as in NF1, it results in extended RAS/mitogen-activated protein kinase (MAPK) downstream signaling pathway activity and, ultimately, uncontrolled cellular proliferation [9-11].

The development of breast cancer in patients with NF1 is a rare occurrence. The connection between the presence of breast cancer (BRCA) 1 and BRCA 2 genes and an increased risk of hereditary breast cancer has been well established. Interestingly, a link between NF1 and breast cancer was suggested because both genes are in very close proximity to each other on the long arm of chromosome 17 and it was thought that a mutation in one could affect the other [12].

Very few cases of NF1 and PTC have been documented in the literature so far [4,5,6,13]. The genetic mechanism characterizing the association between NF1 and PTC is not fully understood. In PTC, even though no documented germ-line mutations have been reported, mutations in RAS proteins have been associated with cancer phenomenon [14]. Newly discovered variations in B type Raf kinase (BRAF) have been shown to have a critical role in PTC tumorigenesis. This occurs via activation of the MAPK pathway, similar to RAS and RET/PTC, indicating a genetic susceptibility of developing PTC via more than one molecular mechanism [14,15]. Moreover, increased ER and PR expression in thyroid tissues have been reported in patients with thyroid cancer [16,17].

Another interesting mechanism for genetic susceptibility has been described through mutations in phosphatase and tensin homolog (PTEN), succinate dehydrogenase (SDHx), and killin (KLLN) genes. Alterations in these genes have been reported to cause Cowden syndrome, which has been proven to result in a higher risk for both breast and thyroid cancer in the same individual [18,19].

## Conclusions

Metachronous malignancies, as described in this case, are infrequent occurrences in oncology. This case underscores the molecular basis of breast and thyroid tumorigenesis in patients with NF1. As a prior history of either cancer has been shown to increase the risk of the other, physicians should strive to identify the association between breast and thyroid cancers. This higher risk of a second primary malignancy could be owed to genetic propensity, hormonal overexpression, or a combination of both. Further studies on a larger scale are needed to determine criteria to screen for primary cancers in patients with NF1, which could help to reduce the disease burden and hopefully improve clinical outcomes.
